# Safety and efficacy of canakinumab treatment for undifferentiated autoinflammatory diseases: the data of a retrospective cohort two-centered study

**DOI:** 10.3389/fmed.2023.1257045

**Published:** 2023-11-15

**Authors:** Ekaterina Alexeeva, Meiri Shingarova, Tatyana Dvoryakovskaya, Olga Lomakina, Anna Fetisova, Ksenia Isaeva, Aleksandra Chomakhidze, Kristina Chibisova, Elizaveta Krekhova, Aleksandra Kozodaeva, Kirill Savostyanov, Aleksandr Pushkov, Ilya Zhanin, Dmitry Demyanov, Evgeny Suspitsin, Konstantin Belozerov, Mikhail Kostik

**Affiliations:** ^1^Department of Pediatric Rheumatology, National Medical Research Center of Children's Health, Moscow, Russia; ^2^Clinical Institute of Children's Health named after N.F. Filatov, Chair of Pediatrics and Pediatric Rheumatology of the Sechenov First Moscow State Medical University, Sechenov University, Moscow, Russia; ^3^Department of Medical Genetics of the Medical and Genetic Center, National Medical Research Center of Children's Health, Moscow, Russia; ^4^Department of Medical Genetics, Saint-Petersburg State Pediatric Medical University, Saint-Petersburg, Russia; ^5^Department of Tumor Growth Biology, N.N. Petrov National Research Center of Oncology, Saint-Petersburg, Russia; ^6^Hospital Pediatry, Saint-Petersburg State Pediatric Medical University, Saint-Petersburg, Russia

**Keywords:** autoinflammation, autoinflammatory disorders, AID, interleukin-1, canakinumab, undifferentiated autoinflammatory disorders

## Abstract

**Introduction:**

The blockade of interleukine-1 (anakinra and canakinumab) is a well-known highly effective tool for monogenic autoinflammatory diseases (AIDs), such as familial Mediterranean fever, tumor necrosis factor receptor-associated periodic syndrome, hyperimmunoglobulinaemia D syndrome, and cryopyrin-associated periodic syndrome, but this treatment has not been assessed for patients with undifferentiated AIDs (uAIDs). Our study aimed to assess the safety and efficacy of canakinumab for patients with uAIDs.

**Methods:**

Information on 32 patients with uAIDs was retrospectively collected and analyzed. Next-generation sequencing and Federici criteria were used for the exclusion of the known monogenic AID.

**Results:**

The median age of the first episode was 2.5 years (IQR: 1.3; 5.5), that of the disease diagnosis was 5.7 years (IQR: 2.5;12.7), and that of diagnostic delay was 1.1 years (IQR: 0.4; 6.1). Patients had variations in the following genes: *IL10, NLRP12, STAT2, C8B, LPIN2, NLRC4, PSMB8, PRF1, CARD14, IFIH1, LYST, NFAT5, PLCG2, COPA, IL23R, STXBP2, IL36RN, JAK1, DDX58, LACC1, LRBA, TNFRSF11A, PTHR1, STAT4, TNFRSF1B, TNFAIP3, TREX1*, and *SLC7A7*. The main clinical features were fever (100%), rash (91%; maculopapular predominantly), joint involvement (72%), splenomegaly (66%), hepatomegaly (59%), lymphadenopathy (50%), myalgia (28%), heart involvement (31%), intestinal involvement (19%); eye involvement (9%), pleuritis (16%), ascites (6%), deafness, hydrocephalia (3%), and failure to thrive (25%). Initial treatment before canakinumab consisted of non-biologic therapies: non-steroidal anti-inflammatory drugs (NSAID) (91%), corticosteroids (88%), methotrexate (38%), intravenous immunoglobulin (IVIG) (34%), cyclosporine A (25%), colchicine (6%) cyclophosphamide (6%), sulfasalazine (3%), mycophenolate mofetil (3%), hydroxychloroquine (3%), and biologic drugs: tocilizumab (62%), sarilumab, etanercept, adalimumab, rituximab, and infliximab (all 3%). Canakinumab induced complete remission in 27 patients (84%) and partial remission in one patient (3%). Two patients (6%) were primary non-responders, and two patients (6%) further developed secondary inefficacy. All patients with partial efficacy or inefficacy were switched to tocilizumab (*n* = 4) and sarilumab (*n* = 1). The total duration of canakinumab treatment was 3.6 (0.1; 8.7) years. During the study, there were no reported Serious Adverse Events (SAEs). The patients experienced non-frequent mild respiratory infections at a rate that is similar as before canakinumab is administered. Additionally, one patient developed leucopenia, but it was not necessary to stop canakinumab for this patient.

**Conclusion:**

The treatment of patients with uAIDs using canakinumab was safe and effective. Further randomized clinical trials are required to confirm the efficacy and safety.

## Introduction

Autoinflammatory diseases (AIDs) represent a group of disorders characterized by recurrent episodes of seemingly unprovoked systemic inflammation predominantly mediated by the innate immune system's cells, without the high-titer autoantibodies or antigen-specific T cells that are usually detected in classic autoimmunity (1). Interleukine-1 hyperproduction is a key point in the pathogenesis of AIDs, and interleukine-1 blockade is a successful treatment for known monogenic AIDs, such as familial Mediterranean fever (FMF), cryopyrin-associated periodic syndrome (CAPS), tumor necrosis factor-associated periodic syndrome (TRAPS), and mevalonate kinase deficiency (MKD) syndrome (2–5). More than 50% of patients with AIDs do not have an exact molecular diagnosis (6). These patients exhibit episodes of fever and systemic inflammation, but they do not have the known abovementioned monogenic AIDs; hence, they belong to the family of undifferentiated AIDs (uAIDs) (7). Patients with uAIDs may experience arthralgia, arthritis, myalgia, abdominal pain, skin and mucosal involvement, and fatigue. Due to multiple genetic variants operating in the innate system regulation found in patients with uAIDs, differences are suspected in the pathogenesis of these diseases (8).

A previous study investigating the efficacy of anakinra (an IL-1 receptor antagonist) on uAIDs revealed its efficacy in patients with uAIDs, but the data on canakinumab are scarce and limited to case reports (7, 9). Our study aimed to evaluate the safety and efficacy of canakinumab (anti-IL-1β agent) for patients with uAIDs.

## Methods

### Study design and patients

We included information about 32 patients: 13 (41%) boys and 19 (59%) girls with a median age of 2.5 (0.08; 16.7) years and with uAIDs. The patients were recruited from two tertiary centers in the retrospective cohort study. The study included patients who received the first dose of canakinumab from 2013 to 2022.

### Inclusion criteria

- Patients with uAIDs were included in this study. The term uAIDs includes children who exhibit clear features of an AID but do not fit a specific diagnosis due to either a non-diagnostic phenotype or negative genetic tests that are typical for known AIDs (9). We applied Federici criteria to exclude well-defined monogenic AIDs (10).
- Patients with NGS (with or without variations in genes, operating in autoinflammatory conditions), except for known diseases (FMF, MKD, CAPS, and TRAPS), were included.
- All included patients had received at least one dose of canakinumab.

### Genetic analysis

Genomic DNA was isolated from whole blood samples using a DNA Blood Mini Kit (QIAGEN, Germany), on a QIAcube automated station (QIAGEN, Germany) according to the manufacturer's protocol. The DNA quality and quantity were evaluated spectrophotometrically on a NanoPhotometer N60 (Implen, Germany) and using a Qubit dsDNA HS Assay Kit for a Qubit 3.0 fluorometer (Invitrogen, USA). Genomic libraries were prepared using the KAPA library preparation kit (Rosche USA). Sequencing was performed using custom NGS panels, containing 14 and 83 genes. Additionally, clinical or whole exome sequencing was performed for three patients. Enrichment was carried out using SeqCapEZ technology (Roche, USA). The total size of the panel was 50 and 200 kbp, while the average reading depth was 100×. The number of reads with 50× depth was more than 99% in all target areas. Miseq (Illumina, USA) was used as a sequencing platform. Bioinformatic analysis was carried out according to the recommendations of GATK Best Practices (https://gatk.broadinstitute.org/).

All detected genome missense variants with a frequency of <5% according to the international base gnomAD version 2.1.1 (http://gnomad.broadinstitute.org), which were absent from the HGMD prof database (version 2020.1., June 2020), underwent bioinformatic analysis. Consequently, we separated variants with pathogenicity confirmed by at least three out of four bioinformatic resources: SIFT (damaging), PolyPhen-1 (probably damaging), PolyPhen-2 (probably damaging), and Mutation Tester (disease-causing). The conserved novel missense and splicing mutations were analyzed using the bioinformatic software Alamut Visual Plus (version 1.5.1).

### Exclusion criteria

Patients with known rheumatic conditions, infections, malignancies, confirmed primary immunodeficiency syndromes, known monogenic AIDs (FMF, TRAPS, MKD, CAPS), or other known causes of periodic fever unrelated to AIDs were excluded from the study. Patients with PFAPA syndrome were also not included in the study.

### Assessment and outcomes

For every patient, we evaluated the following parameters:

- Demography: We evaluated the onset age, age of genetic testing, gender, family history, diagnosis delay, and initial diagnosis.
- Clinical characteristics: We evaluated the fever duration and number of fever episodes per year, the presence of rash and its characteristics, the presence of lymphadenopathy, hepatomegaly, splenomegaly, arthritis/arthralgia, myalgia, eye, ear, and CNS involvement. For patients with persistent fever, the number of fever episodes was established as 12 per year.
- Laboratory findings: We evaluated the C-reactive protein (CRP), erythrocyte sedimentation rate (ESR), white blood cells (WBC), platelets, and the hemoglobin level during the acute phase of the disease.
- Data of observed genetic variants classified according to the ACMG criteria: We classified the data into pathogenic, likely pathogenic, variant of unknown significance, likely benign, and benign (11).
- Treatment before canakinumab administration and their efficacy, time since disease onset, and time between the diagnosis and canakinumab administration were evaluated.
- Characteristics of canakinumab treatment: We evaluated the characteristics of canakinumab treatment, including initial doses and frequency.
- Outcomes of canakinumab treatment: We evaluated the outcomes, including remission (complete, partial, or no remission) and time before remission. In cases of failed efficacy, the type of inefficacy (primary or secondary) was evaluated, as well as the switching to other biologic drugs. The duration of canakinumab treatment was also evaluated.
- Complete remission indicates the absence of clinical [physician global assessment (PGA) = 0/2] and laboratory features (normal CRP) of the disease without corticosteroid treatment. Partial remission indicates significant clinical and laboratory improvement according to the attending physician's opinion or clinical remission with remaining laboratory activity.

#### The study's endpoints, outcomes, and assessments

The primary aim was to assess the efficacy of canakinumab by the type of response, and its corresponding primary endpoint would be the number/percentage of patients achieving complete and/or partial response alongside their definitions.

- Complete response was defined as clinical and serological remission, as well as normal CRP.
- Partial response was defined clinically as a change in the Likert category to a category below and/or serologically as a 50% reduction in CRP but not in the normal range (0–5 mg/l) (7, 12).
- No response included patients who failed to meet the criteria for either remission or partial response (as above), despite canakinumab.

The secondary aims were paired comparisons of (i) markers of systemic inflammation before and after the treatment (ESR, CRP, WBC, PLT, and hemoglobin); (ii) concomitant treatment changes, including steroids-sparing effect; and (iii) the safety profile.

The data on adverse events were collected during routine follow-up visits, by asking parents or looking at patients' medical papers.

#### Statistics

The sample size was not calculated. The software XLSTAT Version 2016.02.28451, Addinsoft, France (release 10.0, StatSoft Corporation, Tulsa, OK, USA), was used for data analyzes. The descriptive statistics were reported in medians and interquartile ranges (IQRs) for continuous variables and in absolute frequencies and percentages for categorical variables. A comparison of quantitative variables recorded before and after therapy was carried out using the Wilcoxon criterion, and a similar comparison of categorical variables was carried out using the McNemar criterion. Survival analysis of each group, with each being flare-free as the event of interest, was conducted by employing the Kaplan–Meier method. The log-rank test was performed to compare the survival curves. The differences were considered statistically significant at a *p*-value of < 0.05.

## Results

### Patients' characteristics at the disease onset

The median age of disease onset was 2.5 (1.3; 5.5) years, that of disease diagnosis was 5.7 (2.5; 12.7) years, and that of diagnostic delay was 1.1 (0.4; 6.1) years. The main clinical features were fever (100 %) and rash (91%), which included maculopapular rash predominantly (53.3%), polymorphic rash (16.7%), erythematous rash (10%), coarse-spotted and finely-spotted rash (7%), and papular and urticaria rash (3%). Joint involvement occurred in 72% of the patients had polyarticular (54%) and oligoarticular (46%) courses. Musculoskeletal manifestations were mainly represented by arthralgia (96%), arthritis (83%), reduced range of motion (79%), and myalgia (28%). Splenomegaly was noted in 66% of the patients, hepatomegaly in 59%, and lymphadenopathy in 50% of the patients. Serositis was detected in 17 patients, with pericardial effusion in 10 (31%) of them, followed by pleural (*n* = 5; 16%) and abdominal effusion (*n* = 2; 6%).

Four patients (9%) had eye involvement, including conjunctivitis (*n* = 2; 6%) and congenital cataract (*n* = 1; 3%). Six patients (19%) had gastrointestinal involvement, and eight patients (25%) experienced the failure to thrive. One patient (3%) had sensorineural hearing loss, and one patient (3%) had hydrocephalus. The highest parameters of laboratory activity at the disease onset were as follows: ESR 47 mm/h (30; 58) and CRP 75.6 mg/l (42.5; 119.4), white blood cells 18.5 (13; 21.4) × 10^9^/l, with neutrophilia 12.6 (17; 52.8) × 10^9^/l, thrombocytosis 449 (320.5; 529) × 10^9^/l, and anemia: hemoglobin 93 (85; 102) g/l. The data are presented in Table 1.

**Table 1:** Initial characteristics of the patients with uAIDs.

**Features**	**Patients (*n =* 32)**
**Demography**	
Gender, women, n (%)	19 (59)
Onset age, years, Me (25%; 75%)	2.5 (1.3; 5.5)
Age of the diagnosis, years, Me (25%; 75%)	5.7 (2.5; 12.7)
Diagnostic delay, years, Me (25%; 75%)	1.1 (0.4; 6.1)
**Clinical features**	
Fever, *n* (%)	32 (100)
Rash, *n* (%)	29 (91)
Joint damage, *n* (%)	23 (72)
Splenomegaly, *n* (%)	21 (66)
Hepatomegaly, *n* (%)	19 (59)
Lymphadenopathy, *n* (%)	16 (50)
Pericarditis, *n* (%)	10 (31)
Myalgia, *n* (%)	9 (28)
Failure to thrive, *n* (%)	8 (25)
Intestine damage, *n* (%)	6 (19)
Pleurisy, *n* (%)	5 (16)
Eye damage, *n* (%)	3 (9)
Ascites, *n* (%)	2 (6)
Sensorineural hearing loss, *n* (%)	1 (3)
Hydrocephalus, *n* (%)	1 (3)
**Laboratory features (onset)**	
Erythrocyte sedimentation rate, mm/h, Me (25%; 75%)	47 (30; 58)
C-reactive protein, mg/l Me (25%; 75%)	75.6 (42.5; 119.4)
White blood cells, 10^9^/l, Me (25%; 75%)	18.5 (13; 21.4)
Neutrophils, 10^9^/l, Me (25%; 75%)	12.6 (17; 52.8)
Platelets, 10^9^/l, Me (25%; 75%)	449 (321; 529)
Hemoglobin, g/l, Me (25%; 75%)	93 (84.5; 102)
**Laboratorial (canakinumab treatment)**	
Erythrocyte sedimentation rate, mm/h, Me (25%; 75%)	3 (2; 7)
C-reactive protein, mg/l Me (25%; 75%)	0.7 (0.25; 1.9)
White blood cells, 10^9^/l, Me (25%; 75%)	6.6 (5.6; 8.9)
Neutrophils, 10^9^/l, Me (25%; 75%)	3.1 (2.2; 3.7)
Platelets, 10^9^/l, Me (25%; 75%)	268 (226; 341)
Hemoglobin, g/l, Me (25%; 75%)	124 (119; 134)
**Treatment using canakinumab (*****n** =* **32)**	
Age during canakinumab administration, years, Me (25%; 75%)	6.1 (3.6; 11)
Time from disease onset to canakinumab administration, years, Me (25%; 75%)	2.5 (0.6; 5.8)
Time from diagnosis to canakinumab administration, years, Me (25%; 75%)	0.35 (0.1; 2.6)
Duration of canakinumab treatment, years, Me (25%; 75%)	3.5 (1.3; 5.2)
**Outcomes**	
Complete remission, *n* (%)	27 (84)
Partial remission, *n* (%)	1 (3)
Non-responders, *n* (%)	4 (12)
Inefficacy primary, *n* (%)	2 (6)
Inefficacy secondary, *n* (%)	2 (6)
Adverse events (leucopenia), *n* (%)	1 (3)

Me, median.

### Genetic testing

Next-generation sequencing was performed in all patients, and no genetic variants were found only in two (6%) patients. One patient underwent clinical exome sequencing, and two patients underwent whole-exome sequencing. NGS was performed using a panel of 15 genes in six patients (18%) and using a panel of 83 genes in 24 patients (73%). Clinical exome sequencing was performed in one patient (3%), and whole-exome sequencing was performed in two patients (6%). Heterozygous genetic variants were found in the remaining 31 patients (97%) in the following genes: *IL10, NLRP12, STAT2, C8B, LPIN2, NLRC4, PSMB8, PRF1, CARD14, IFIH1, LYST, NFAT5, PLCG2, COPA, IL23R, STXBP2, IL36RN, JAK1, DDX58, LACC1, LRBA, TNFRSF11A, PTHR1, STAT4, TNFRSF1B, TNFAIP3*, and *TREX1* и *SLC7A7*. According to the ACMG criteria, pathogenic variants were found in the genes *C8B* (*p*.*R428X*) and *TREX1* (*p.Y360C*), likely pathogenic variants were found in the gene *NFAT5* (*p.A622V*), likely benign variants were found in the genes *PSMB8* (*p.G8R*), *STAT2* (*p.G825C*), *TNFRSF1B* (*p.P205A*) and *IL23R* (*p.G149R*), and variants of unknown significance were found in the genes *IL10* (*p.G15R*), *PTHR1* (*p.R150C*), and *STAT4* (*p.E128V*). Variants with unknown significance were the most frequent, accounting for 35 out of 45 variants (77.8%) among the total identified variants. In our study, patients neither met the Federici criteria nor did they have variants in the *NLRP3, TNFRSF1A, MVK*, and *MEFV* genes according to the NGS data. When applying the Federici criteria, the majority of patients (*n* = 29, 91%) from our study were not clinically classified into any of the main syndromes of periodic fever. Table 2 lists the genetic mutations detected for each patient.

**Table 2:** Genetic findings in the studied population.

**Gene, transcript**	**Inheritance**	**HGVS coding and protein**	**Minor allele frequency (gnomAD Exomes v.2.1.1)**	**References**
*IL10* NM_000572.3	No data	*c.43G>A, p.G15R*	0.2%	(13, 14)
*NLRP12* NM_144687.4	AD	*c.910C>T, p.H304Y*	0.4%	(15, 16)
		*c.1316T>A, p.L439Q*	0.06%	(17)
		*c.1659G>C, p.R553S;*	No data	-
		*c.1054C>T, p.R352C*	0.04%	(16, 18)
*STAT2* NM_005419.4	AR	*c.2473G>T, p.G825C*	1.6%	(19, 20)
*C8B* NM_000066.4		c.1282C>T, R428^*^	0.1%	(21, 22)
*LPIN2* NM_001375808.2	AR	*c.1489G>A, p.E497K*	0.03%	(23, 24)
		*c.2621G>T, p.C874F*	0.1%	(25, 26)
		*c.1510C>T, p.L504F*	0.3%	(27, 28)
		*c.1814C>T, p.S605L*	0.002%	(29)
		*c.1456 + 4C>G*	0.01%	-
*NLRC4* NM_001199138.2	AD	*c.1550G>C, p.C517S;*	0.003%	-
		c.928C>T, p.R310^*^	0.02%	(30, 31)
*PSMB8* NM_148919.4	AR	*c.22G>A, p.G8R*	0.02%	(32, 33)
		*c.392T>C, p.L131P*	0.0004%	-
		*c.220_222delinsTCA, p.T74S*	Absent	(32)
*PRF1* NM_001083116.3	AR	*c.368G>A, p.R123H*	0.05%	(34, 35)
*CARD14* NM_001366385.1	AD	*c.1772C>T, p.T591M*	0.07%	(36, 37)
*IFIH1* NM_022168.4	AD, AR	*c.1865C>T, p.A622V*	0.005%	(25, 38)
		*c.229C>T, p.R77W*	0.07%	(20, 39)
*LYST* NM_000081.4	AR	*c.7385C>A, p.A2462E*	0.04%	(40)
		*c.6710A>C, p.Q2237P*	0.1%	(40, 41)
		*c.7870C>T, p.R2624W*	0.3%	(25, 42)
*NFAT5* NM_138713.4	No data	*c.74-1G>C*	No data	-
*PLCG2* NM_002661.5	AD	*c.1506G>C, p.K502N*	No data	-
		*c.1063T>A, p.C355S*	No data	-
*COPA* NM_004371.4	AD	*c.1531A>G, p.I511V*	No data	-
*IL23R* NM_144701.3	No data	*c.445G>A, p.G149R*	0.7%	(43, 44)
*STXBP2* NM_001272034.2	AR	*c.398G>A, p.R133H*	0.06%	(45, 46)
*IL36RN* NM_012275.3	AR	*c.368C>T, p.T123M*	0.004%	(47, 48)
*JAK1* NM_002227.4	AD	*c.656G>A, p.R219Q*	0.002%	(38)
*DDX58* NM_014314.4	AD	*c.109G>A, p.E37K*	0.0008%	(49)
*LACC1* NM_153218.4	AR	*c.632A>G, p.N211S*	0.0008%	-
		*c.988_990del, p.I330del*	0.0008%	(50, 51)
*LRBA* NM_001364905.1	AR	*c.349A>G, p.I117V*	0.002%	(52, 53)
*TNFRSF11A* NM_003839.4	AD, AR	*c.718A>G, p.K240E*	0.1%	(54, 55)
*PTHR1* NM_000316.3	AD, AR	*c.448C>T, p.R150C*	0.2%	(56, 57)
*STAT4* NM_003151.4	No data	*c.383A>T, p.E128V*	0.1%	(58, 59)
*TNFRSF1B* NM_001066.3	No data	*c.613C>G, p.P205A*	0.2%	(60, 61)
*TNFAIP3* NM_001270508.2	AD	*c.1217A>T*, p.E406V	No data	-
*TREX1* NM_033629.6	AD, AR	*c.914A>G, p.Y305C*	0.01%	(62, 63)
*SLC7A7* NM_003982.4	AR	*c.1400A>T, p.K467M*	0.3%	(60)

^*^old name; nd, no data; VUS, variant of unknown significance.

### Initial treatment and canakinumab administration

The initial treatment before canakinumab consisted of non-biologic drugs: non-steroidal anti-inflammatory drugs (NSAID) (91%), corticosteroids (85%), methotrexate (38%), intravenous immunoglobulin (IVIG) (34%), cyclosporine A (24%), colchicine (6%), cyclophosphamide (6%), sulfasalazine (3%), and mycophenolate mofetil (3%), and biologic drugs: tocilizumab (62%), sarilumab, etanercept, adalimumab, rituximab, and infliximab (all by 3%). Before the year 2022, we had no access to anakinra. The median time from the disease onset to canakinumab administration was 2.5 (1.3; 5.5) years. Thirteen patients (41%) received canakinumab as the first-line biologic treatment, and 19 patients (59%) received it as the second-line biologic treatment. In the majority of patients, 26 (81%), the initial dose of canakinumab was 4 mg/kg every 4 weeks, in 5 patients (16%), it was 2 mg/kg every 4 weeks, and in one patient (3%), it was 8 mg/kg every 4 weeks. The detailed patients' characteristics are presented in Table 3.

**Table 3:** Detailed characteristics of patients with uAIDs treated with canakinumab.

		**Results of NGS**	**Clinical features**													
**ID**	**Gender**		**Fever**	**Rash**	**Serosites**	**Arthritis**	**GI**	**Eye**	**Hydrocephalus**	**Sensorineural hearing loss**	**Laboratory activity**	**Onset age**	**Age at the time of diagnosis**	**Initial treatment (non-biologic)**	**Initial treatment (biologic drugs) **	**Outcome**
1	W	*NLRC4* {c.928 C>T, p.R310X}	**+**	**+**	**-**	**+**	**-**	**-**	**-**	**-**	**+**	1.2	3.6	Colchicine NSAID	CAN	CR
2	W	*PSMB8* {c.22 G>A, p.G8R}	**+**	**+**	**-**	**+**	**-**	**-**	**-**	**-**	**+**	1.6	11.6	GCS, MTX, CsA, NSAID	TCZ, CAN	CR
3	M	*LPIN2* {c.1489 G>A, p.E497K}	**+**	**+**	**-**	**+**	**+**	**-**	**-**	**+**	**+**	1.9	3.5	NSAID	CAN	CR
4	W	*LPIN2* {c.2621 G>T, p.C874F}	**+**	**+**	**+**	**+**	**-**	**+**	**-**	**-**	**+**	1.4	2.5	GCS, IVIG, NSAID	CAN TCZ	NR (PI)
5	W	*LPIN2* {c.1510C>T, p.L504F}	**+**	**+**	**+**	**+**	**-**	**-**	**-**	**-**	**+**	3.9	15	GCS, IVIG, MTX, CTX, CsA, NSAID	RTX, ADA INF, TCZ, CAN	CR
6	M	*PSMB8* {c.392C>T, p.L131P}	**+**	**+**	**-**	**+**	**-**	**-**	**-**	**-**	**+**	5.6	5.7	GCS, IVIG, MTX, CsA, NSAID	TCZ, CAN	CR
7	M	*STAT4* {c.383A>T, p.E128V}	**+**	**+**	**+**	**+**	**-**	**-**	**-**	**-**	**+**	1.4	1.7	GCS, NSAID	TCZ, CAN	CR
		*TNFRSF1B* {c.613C>G, p.P205A}														
8	W	*PSMB8* {c.220_222delinsTCA, p.T74S}	**+**	**+**	**-**	**+**	**-**	**-**	**-**	**-**	**+**	2.5	3.9	GCS, MTX, NSAID	TCZ, CAN	CR
		*PRF1* {c.368G>A, p.R123H}														
9	W	*IL23R* {c.445G>A (heterozygous), p.G149R}	**+**	**+**	**-**	**+**	**-**	**-**	**-**	**-**	**+**	1.5	2.3	GCS, CsA, NSAID	TCZ, CAN	CR
		*STXBP2* {c.398G>A, p.R133H}														
10	M	*NLRP12* {c.910C>T, p.H304Y}	**+**	**+**	**+**	**+**	**-**	**-**	**-**	**-**	**+**	4.5	15.5	GCS, MTX, CTX, NSAID	TCZ, CAN	CR
		*IL10* {c.43G>A, p.G15R}														
11	W	*PLCG2* {c.1506G>C, p.K502N}	**+**	**+**	**+**	**-**	**-**	**-**	**-**	**-**	**+**	3.4	4	GCS, MTX, SSZ	TCZ, CAN ANA, SAR	NR (SI)
12	M	*TREX1* {c.1079A>G, p.Y360C}	**+**	**+**	**-**	**+**	**-**	**-**	**-**	**-**	**+**	4.1	4.4	GCS, IVIG, NSAID	TCZ, CAN	CR
		*SLC7A7* {c.1400A>T, p.K467M}														
13	W	*NLRP12* {c.0.1316T>A, p.L439Q}	**+**	**+**	**-**	**+**	**+**	**-**	**-**	**-**	**+**	0.8	1.5	GCS, IVIG CsA	TCZ, CAN	CR
14	M	*PTHR1* {c.448C>T, p.R150C}	**+**	**+**	**-**	**+**	**-**	**-**	**-**	**-**	**+**	1.2	5.7	GCS, IVIG, MTX, MMF	CAN	CR
15	W	*IFIH1* {c.1865C>T, p.A622V}	**+**	**+**	**-**	**+**	**-**	**+**	**-**	**-**	**+**	2.6	2.8	GCS, NSAID	TCZ, CAN	CR
16	W	*NFAT5* {c.74-1G>C, p.A622V}	**+**	**+**	**-**	**+**	**-**	**-**	**-**	**-**	**+**	6.5	6.8	GCS, IVIG NSAID	TCZ, CAN	CR
		*LYST* {c.7870C>T, p.R2624W}														
17	W	*TREX1* {c.1079A>G, p.Y360C}	**+**	**+**	**+**	**+**	**+**	**-**	**-**	**-**	**+**	5.3	7.7	GCS, MTX, NSAID	TCZ, CAN	CR
18	W	*NLRC4* {c.1550 G>C, p.C517S}	**+**	**+**	**-**	**+**	**-**	**-**	**-**	**-**	**+**	1.3	2.5	GCS, IVIG, MTX, NSAID	TCZ, CAN	CR
		*LPIN2* {c.1456 + 4C>G, p. -}														
19	W	*LPIN2* {c.1814C>T, p.S605L	**+**	**+**	**+**	**-**	**-**	**-**	**-**	**-**	**+**	10.8	11	GCS, IVIG, CsA, NSAID	TCZ, CAN	CR
20	M	*IFIH1* {c.229C>T, p.R77W}	**+**	**+**	**-**	**+**	**-**	**-**	**-**	**-**	**+**	2.5	3.3	GCS, MTX NSAID	TCZ, CAN	CR
		*LYST* {c.7385C>A, p.A2462E}														
21	W	*LYST* {c.7385C>A, p.A2462E}	**+**	**+**	**+**	**-**	**-**	**-**	**+**	**-**	**+**	16.7	17.2	GCS, IVIG, CsA, HCQ NSAID	CAN	CR
		*LYST* {c.c.6710A>C, p.Q2237P}														
22	M	*LACC1* {c.632A>G, p.N211S}	**+**	**+**	**-**	**+**	**+**	**-**	**-**	**-**	**+**	1.1	1.8	GCS, MTX, NSAID	TCZ, CAN	CR
		*LACC1* {c.988_990del, p.I330del}														
23	W	*COPA* {c.1558A>G, p.I520V}	**+**	**+**	**-**	**+**	**-**	**-**	**-**	**-**	**+**	1.9	2.3	GCS, NSAID	TCZ, CAN, TOF, SAR	NR (PI)
24	W	*NLRP12* {c.1659G>C, p.R553S}	**+**	**+**	**-**	**+**	**-**	**+**	**-**	**-**	**+**	7.8	8.7	GCS, NSAID	TCZ, SAR, CAN	CR
25	W	*PLCG2* {c.1063T>A, p.C355S}	**+**	**+**	**+**	**+**	**-**	**-**	**-**	**-**	**+**	4	16.8	GCS, IVIG, MTX, CsA	ETA, TCZ, CAN	CR
26	M	*TNFAIP3* {c.1217A>T, p.E406V}	**+**	**-**	**-**	**+**	**-**	**-**	**-**	**-**	**+**	5.7	14.7	GCS, NSAID	TCZ, CAN	CR
27	M	LRBA {c.349A>G, p.I117V}	**+**	**-**	**-**	**-**	**-**	**-**	**-**	**-**	**+**	1.4	13.8	GCS, NSAID	CAN	CR
		*TNFRSF11A* {c.718A>G, p.K240E}														
28	M	-	**+**		**-**	**-**	**+**	**-**	**-**	**-**	**+**	0.3	0.5	GCS, NSAID	CAN, TCZ	PR
29	W	-	**+**	**+**	**-**	**-**	**-**	**-**	**-**	**-**	**+**	3	23.4	NSAID	CAN	CR
30	M	*JAK1* {c.656G>A, p.R219Q}	**+**	**-**	**+**	**-**	**-**	**-**	**-**	**-**	**+**	5.9	7.4	GCS, Colchicine	CAN, TCZ	NR (SI)
		*DDX58* {c.109G>A, p.E37K}														
31	M	*IL36RN* {c.0.368C>T, p.T123M}	**+**	**+**	**-**	**-**	**-**	**-**	**-**	**-**	**+**	0.1	7.7	GCS, NSAID	CAN	CR
32	W	*NLRP12* {c.1054C>T, p.R352C}	**+**	**+**	**-**	**-**	**-**	**-**	**-**	**-**	**+**	0.2	0.3	NSAID	CAN	CR
		*C8B* {c.1282C>T, R428^*^}														

ADA, adalimumab; ANA, anakinra; CAN, canakinumab; CR, complete remission; CsA, cyclosporine A; CTX, cyclophosphamide; GCS, corticosteroids; ETA, etanercept; HCQ, hydroxychloroquine; INF, infliximab; IVIG, intravenous immunoglobulin; MTX, methotrexate; MMF, mycophenolate mofetil; NR, non-responder; NSAID, non-steroidal anti-inflammatory drugs; PI, primary inefficacy; PR, partial remission; RTX, rituximab; SAR, sarilumab; SI, secondary inefficacy SSZ, sulfasalazine; TCZ, tocilizumab; and TOF, tofacitinib.

### Primary endpoint: canakinumab treatment outcomes

Complete remission was achieved in 27 (84%) patients. Two patients (6%) displayed secondary inefficacy in 3 months, while two others displayed primary inefficacy. One patient had a partial response to canakinumab. In all five patients, canakinumab was switched to other drugs: tocilizumab (*n* = 3; 60%), anakinra (*n* = 1; 20%), and tofacitinib (*n* = 1; 20%). In two patients, it was switched to tocilizumab, and complete remission was achieved. The remaining two patients who were switched to anakinra and tofacitinib required a switch to sarilumab administration, which induced remission. One patient (Pt 4) died after switching to tocilizumab. This patient had early onset of symptoms (at 1.5 years), with rash, pericarditis, bilateral interstitial lung disease, and MAS. Unfortunately, the patient failed to respond to high-dose intravenous and oral glucocorticosteroids and IVIG. Canakinumab was prescribed due to MAS with multiorgan failure. The patient partially responded to the first dose (which resulted in decreased fever, rash, arthritis, and laboratory activity) but developed a new flare after an attempt to taper corticosteroid treatment and was switched to tocilizumab. The patient again had a partial response but died due to the exacerbation of the underlying disease, protract MAS, multiorgan failure, and infection complication.

An analysis of the effectiveness depending on the time of initiation of therapy with canakinumab was carried out. Early administration (≤ 12 months from onset) of canakinumab was carried out in 11 out of 32 patients (35%). Consequently, therapy was effective in 9 out of 11 patients (82%), one patient (9%) had primary inefficiency, and one patient (9%) had secondary inefficiency. Late administration of canakinumab (>12 months from onset) was carried out in 21 out of 32 patients (65%). Consequently, therapy was effective in 18 out of 21 patients (85%), one patient had primary inefficiency (5%), one patient developed secondary inefficiency (5%), and one patient had a partial response (5%).

As the first line of therapy, canakinumab was administered early to 5 out of 11 patients (46%) and late to 7 out of 21 patients (33%). It was prescribed as the second line of therapy in 6 out of 11 patients (54%) in the group with early initiation and in 14 out of 21 patients (67%) in the group with late initiation.

**Figure 1:**
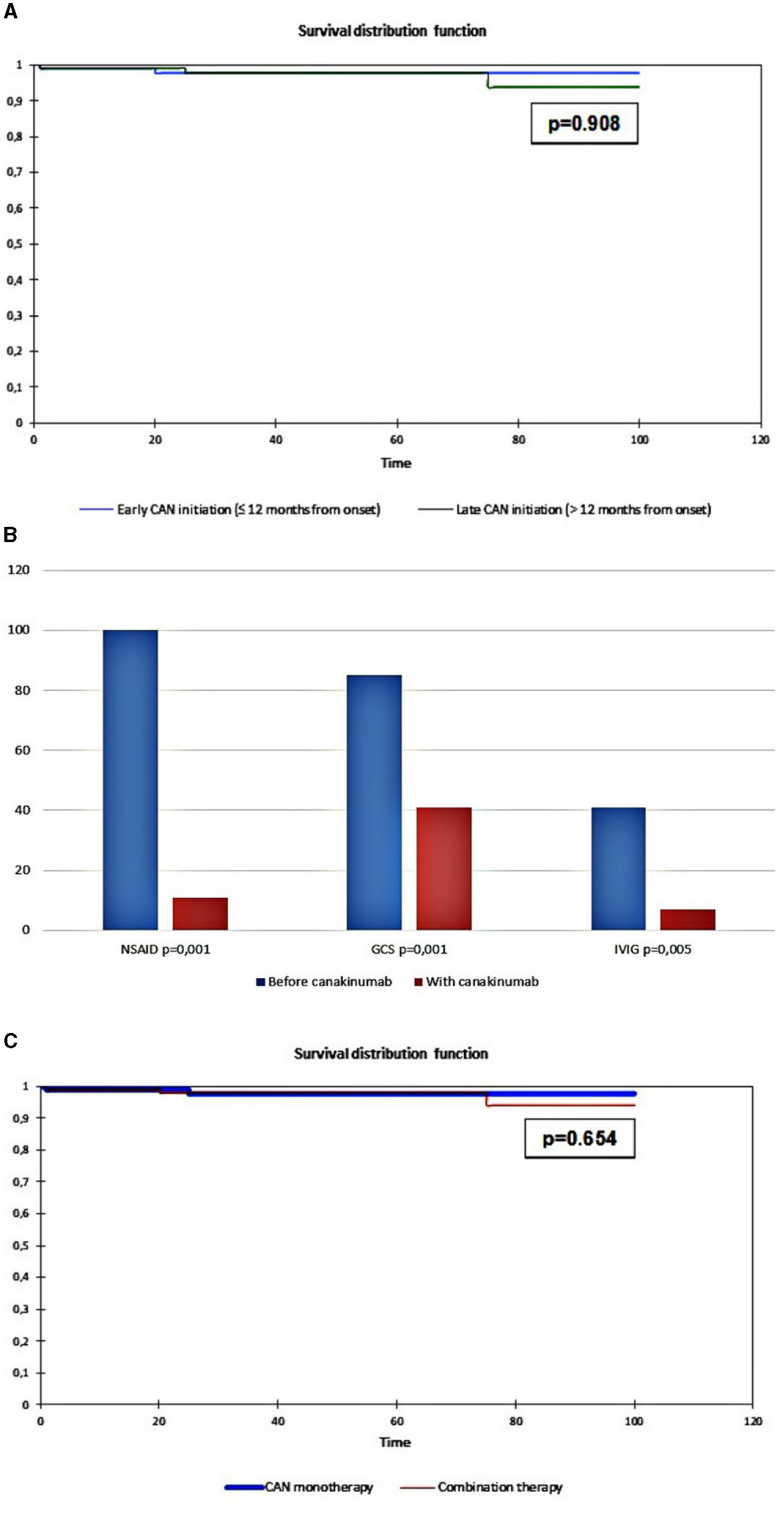
**(A)** Cumulative probability of being flare-free in uAID patients depending on early canakinumab administration (≤12 months from the onset, blue line) and late canakinumab administration (>12 months from the onset, green line); **(B)** Dynamics of concomitant treatment during the canakinumab trial; **(C)** Cumulative probability of being flare-free in uAID patients depending on the canakinumab monotherapy (canakinumab only, blue line) and the combination of canakinumab with non-biologic DMARDs (combination therapy, red line).

Therapy survival was evaluated in 11 out of 32 patients (35%) with early initiation of canakinumab treatment and 21 out of 31 patients (65%) with late initiation of canakinumab treatment. The flare-free survival in the group of patients with early administration was 96.1 months, and that in the group with late administration was 96.2 months (*p* = 0.908; Figure 1A).

### Secondary endpoints

i) *Dynamics of the markers of systemic inflammation*
  During the 12-month canakinumab trial, ESR decreased significantly from 53 (23; 57) mm/h to 3 (2; 7) mm/h (*p* < 0.001), CRP decreased from 73.5 (46; 103) mg/l to 0.7 (0.3; 1.9) mg/l (*p* < 0.001), WBC decreased from 18.6 × 10^9^/l (12.9; 21.8) to 6.6 (5.6;8.9) × 10^9^/l (*p* < 0.001), neutrophil count decreased from 11.6 × 10^9^/l (17.4; 11.6) to 3.1 (2.2; 3.7) × 10^9^/l, (*p* < 0.001), platelets decreased from 439 × 10^9^/l (307; 560) to 268 (226; 341) × 10^9^/l (*p* = 0.003), and hemoglobin increased from 96 g/l (89; 102) to 124 (119; 134) g/l (*p* < 0.001).
ii) *Changes in the concomitant treatment*
  All patients in our study received concomitant treatment. At the onset, 29 patients (91%) received NSAID, 28 patients (88%) received systemic corticosteroids, 12 patients (38%) received methotrexate, 11 patients (34%) received IVIG, 8 patients (25%) received cyclosporine A, 2 patients (6%) received cyclophosphamide, 2 patients (6%) received colchicine, and 1 patient (3%) received sulfasalazine, mycophenolate mofetil, and hydroxychloroquine.
  Among those who responded to canakinumab (*n* = 27), corticosteroids were successfully discontinued in 12 patients (44%, *p* = 0.001), NSAID was discontinued in all patients (100%, *p* = 0.001), and IVIG was discontinued in 9 patients (82%, *p* = 0.005). All other non-biologic medications were also discontinued. Data are shown in Figure 1B.
  Flare-free survival (Figure 1C) was 97.1 months for 17 out of 32 (53%) patients treated with canakinumab monotherapy, and it was 96.1 months for 15 out of 32 (47%) patients treated with combination therapy (canakinumab + non-biologic DMARDS) (Log-rank test, *p* = 0.654).
iii) *Safety profile*
  Regarding the safety profile, no serious adverse events were observed during canakinumab treatment. Five patients developed mild acute respiratory infections, with a similar rate to what was already detected before canakinumab initiation. One case of leukopenia was also reported, but treatment discontinuation was not required.

## Discussion

Canakinumab showed efficacy in patients with uAID, which was realized in the control of clinical and laboratory features of inflammation as well as in the reduction of concomitant immune-suppressive treatment. We used inclusion criteria for these groups, similar to what was published earlier (9). Canakinumab showed efficacy in AIDs in an earlier study, where it was administered off-label (64). It was successfully prescribed in several distinct AIDs, including DIRA, Majeed syndrome, PAPA syndrome, DADA2, Blau syndrome, systemic juvenile idiopathic arthritis (sJIA), MAS, and fibrodysplasia ossificans progressiva (65–74). Among polygenic multifactorial AIDs, canakinumab showed efficacy in pediatric and adult Behçet syndrome, being predominantly effective in eye disease (75, 76). There are several case reports of using canakinumab in idiopathic relapsed pericarditis, but the Italian experience suggests the superiority of anakinra over canakinumab, attributed to the main role of IL-1a over IL-1β (77, 78).

We used the results for NGS to exclude patients with well-defined monogenic AIDs because some such patients might present with a non-classical phenotype. Broad molecular testing of patients with uAIDs with NGS panels may provide a diagnosis. In 50 patients with uAIDs, 100 genetic variants were found (2 per patient, range 0–6), a quarter of which were in genes. These were related to autoinflammation, and only two patients had variants, allowing us to make a definitive diagnosis (mevalonate kinase deficiency syndrome). Two more patients had variations in the *PLCG2* gene, consistent with the clinical phenotype APLAID and PLAID, respectively.

### IL-1 blockers for the treatment of uAIDs

During the canakinumab trial, the majority of our patients (84%) achieved remission. The presence of concomitant non-biologic DMARDs did not influence the cumulative probability of being flare-free. Many previous studies have shown no efficacy of non-biologic DMARDs in patients with uAIDs (7, 79). We did not observe any difference between early and late canakinumab administration, as well as between the first-line and second-line biologic therapy.

Similar to our results with the anti-IL1β blocking agent, a complete response to anakinra (interleukin-1 receptor antagonist) was observed in 5 out of 10 patients (50%) and a partial response in 1 out of 10 patients (10%), while 4 out of 10 patients (40%) were non-responders (9, 80). In the biggest pediatric case series of 22 patients with uAIDs, 8 out of 22 patients (36%) achieved clinical and serological remission, 8 out of 22 patients (36%) had a partial response, and 6 out of 22 patients (28%) were non-responders. A total of 16 out of 22 patients (72%) responded to anakinra. More than half the patients (12/22; 55%) required anakinra dosing up-titration to control their disease activity. This pattern of up-titration is typical for IL-1 blocking agents in the treatment of AIDs (7, 79). Eighteen out of 20 patients (90%) had a normal CRP level within 3 months, and 17 out of 18 patients (94%) had a normal CRP level within 6 months.

In the biggest review of pediatric patients with uAIDs, named syndrome of undifferentiated recurrent fever (SURF), IL-1 antagonists (mainly anakinra) used in 46 patients were effective in 74% of cases, superior to other non-biologic treatment options (79).

Anakinra was effective in 9 out of 11 adult patients with uAIDs within 4–6 weeks after starting the treatment. One patient discontinued therapy due to being an incomplete responder and another due to a severe injection-site reaction (9). Previous treatment with systemic corticosteroids failed in 5 of 11 patients; 6 out of 11 patients had partial control and 3 out of 11 patients had side effects, associated with the chronic use of systemic corticosteroids. Ten patients had 16 courses of ineffective non-biologic DMARDS, and four patients had adverse events. Treatment with anakinra not only controlled inflammation but also allowed for the tapering of corticosteroids in responsive patients who were still alive. It also led to a decrease in deceased patients, as well as non-biologic DMARDs in all responders. Non-responders were successfully switched to the IL-6 receptor antagonist, tocilizumab (9).

Anakinra showed efficacy in controlling inflammation in 8 out of 9 adult patients (89%) with uAIDs and in 5 out of 6 patients (83%) with VEXAS syndrome, who had previously failed to respond to systemic corticosteroid treatment (81).

In one patient from our cohort, canakinumab was chosen for the treatment of both uAIDs and MAS, due to the lack of access to anakinra. Data about canakinumab efficacy in MAS is controversial and limited to several studies (65, 74, 82). In real practice, physicians usually choose anakinra as the first-line biologic treatment for MAS (83). In the randomized clinical trial, the frequency of MAS was two times lower than that in placebo. A recent case series showed the efficacy of a high dose of canakinumab in controlling MAS in sJIA (65).

### Treatment of non-responders to IL-1 blockers

Previous data similar to ours showed that IL-1 blockers, which are theoretically a universal drug for the treatment of patients with AIDs, were not effective in all patients.

The pathogenesis of known autoinflammatory diseases consists in the abnormal activation of inflammatory processes; this leads to hyperproduction of IL-1ß or IL-18, which, in turn, stimulates the production of inflammatory cytokines, such as IL-6 and TNF-α, and promotes the activation of immune cells, such as macrophages and T-cells (84). Thus, IL-6 inhibitors with the ineffectiveness of IL-1 inhibitors can be considered potential methods of treatment for AIDs (85).

The treatment of non-responders is a challenging problem, requiring the use of other medications, such as colchicine, IL-6, Janus-kinase inhibitors, or their combination (79, 86). The question of whom an IL-1 blocking agent should be prescribed to and when it is still open and requires the development of a treat-to-target strategy due to the different response rates in the literature (87).

The “new-old” drug colchicine has shown its efficacy in FMF and is currently being repositioned for use in the treatment of other AIDs, such as adult-onset Still disease (AOSD) and uAIDs, with a 96% maximal efficacy rate (9, 84, 85). Ten of 11 undiagnosed patients with a strong suspicion of autoinflammatory conditions and formal diagnosis in three of them responded well (91%) to empirically prescribed colchicine, IL-1, and IL-6 blocking without Serious Adverse Events (SAEs) (88).

Tocilizumab, an IL-6 blocking biologic, showed efficacy in sJIA (89–91) and is currently being repositioned for use in the treatment of AIDs, but its efficacy in AID is lower than that of IL-1 blockers (92). The use of IL-6 inhibitors as a second-line therapy in patients with monogenic AIDs, such as familial Mediterranean fever and TRAPS, has been described (93, 94). Additionally, data on the effectiveness of tocilizumab in a small cohort of children with a refractory course of undifferentiated AIDs has been previously published (95). In some patients, tocilizumab has been observed to work well, especially in patients with a sJIA-like phenotype (96). In our cohort, 21 out of 32 patients (66%) received tocilizumab as the first-line biologic treatment, but the treatment was discontinued in 20 out of 21 patients (95%) due to the following reasons: primary (*n* = 5, 25%) and secondary (*n* = 7; 35%) inefficacies, a partial response (*n* = 4; 20%), and severe adverse events, including leucopenia in 3 out 20 patients (15%), infusion reaction (*n* = 3; 15%) и, and infectious complication (*n* = 1; 5%). The majority of the patients (*n* = 19; 95%) were switched to canakinumab, and one patient (5%) was switched to sarilumab, followed by canakinumab due to the primary inefficacy of sarilumab. Tocilizumab has been shown to be more effective when employed as a second-line option, after the failure of IL-1 blockers (7, 9, 93, 94).

Janus-kinase inhibitors (tofacitinib and baricitinib) are considered a new promising option for the treatment of uAIDs, including AOSD, sJIA, and aoSD- and sJIA-like diseases (49, 86, 97). Among 37 patients with previously undiagnosed inflammatory cases, increased IFN signaling was found in 19 patients, with 10 exhibiting clinical features typical of type I interferonopathy (98). Different signaling pathways might explain the failure of IL-1 inhibitors in patients with uAIDs. It is necessary to mention JAK-STAT signaling in patients with uAIDs, especially those with clinical features of type I interferonopathy (99).

### Safety

In our cohort, there were no reported SAEs. Patients had non-frequent mild respiratory infections with a rate similar to that before canakinumab; one patient developed leukopenia (3%), but it was not required to stop canakinumab. All patients also had a formal diagnosis of sJIA to qualify for biologic treatment.

The treatment of uAIDs with anakinra was associated with SAEs. The main adverse events were death (3/22, 14%), allogeneic hematopoietic stem cell transplantation (1/22, 5%), injection-site reactions (5/22, 23%), infections (8/22, 36%), and neutropenia (7/22, 32%). Ten patients (46%) had 12 SAE episodes. Three deaths occured, among which one was related to macrophage activation syndrome (*n* = 1), while two were due to multiorgan failure resulting from underlined conditions (*n* = 2). In both cases, the patients received anakinra, which was stopped before their deaths (7).

Another study reported two SAEs. Among nine responders two patients died: 81-year-old patient treated with anakinra for 3 months died from influenza and 42-year-old woman treated with anakinra for eight months died due to progression of her underlying condition (9).

Creating a worldwide network of experts (international registries) who can share their experiences will potentially be useful for overcoming some of the unmet needs of patients with AIDs (100).

### Limitations

The main limitations of this study were related to the retrospective study design. The lack of whole-exome sequence data of all participants might have led to the omission of some genetic variants. There were no specific universal treatments and diagnostic algorithms. The authors did not influence time and indication for either the use of biologics or the dynamics of concomitant treatment, which might influence treatment efficacy, outcomes, and adverse events. The data on side effects might also have been incomplete due to the absence of a universal electronic medical system, which would have allowed for the timely checking of all patient events and records.

## Conclusion

Many cases of uAIDs belong to the family of IL-1-driven diseases. The treatment of patients with uAIDs using canakinumab was safe and effective; it may be recommended as a first-line biologic treatment with or without corticosteroids and non-biologic DMARDs. Molecular diagnostics may help categorize patients into a group of uAIDs, and IL-1 blockers can be considered in cases where the IL-1-mediated pathway is suspected. For non-responders, the use of different treatments, e.g., IL-6 blockade or JAK-STAT inhibitors, is required. More robust, powered, and properly designed studies are warranted to draw firm conclusions regarding the efficacy and safety of canakinumab in uAIDs.

## Data availability statement

Publicly available datasets were analyzed in this study. This data can be found here: https://www.ncbi.nlm.nih.gov/bioproject/PRJNA1012850.

## Ethics statement

The Ethics Committee of National Medical Research Center of Children's Health, Moscow, Russian Federation (protocol number 4 from 22.04.2021). The studies were conducted in accordance with the local legislation and institutional requirements. Written informed consent for participation in this study was provided by the participants' legal guardians/next of kin. Written informed consent was obtained from the participant/patient(s) or their legal guardians/next of kin for the publication of this article.

## Author contributions

EA: Writing—original draft, Conceptualization, Funding acquisition, Methodology, Project administration, Supervision, Writing—review and editing. MS: Formal analysis, Investigation, Methodology, Writing—original draft, Writing—review and editing. TD: Investigation, Methodology, Validation, Writing—review and editing. OL: Data curation, Investigation, Writing—review and editing. AF: Investigation, Methodology, Writing—review and editing. KI: Investigation, Methodology, Writing—review and editing. AC: Investigation, Methodology, Writing—review and editing. KC: Investigation, Writing—review and editing. EK: Investigation, Writing—review and editing. AK: Investigation, Writing—review and editing. KS: Writing—review and editing, Formal analysis, Investigation, Methodology, Resources. AP: Data curation, Formal analysis, Investigation, Methodology, Writing—review and editing. IZ: Formal analysis, Investigation, Methodology, Project administration, Resources, Writing—review and editing. DD: Data curation, Formal analysis, Investigation, Methodology, Software, Writing—review and editing. ES: Data curation, Formal analysis, Investigation, Methodology, Writing—review and editing. KB: Formal analysis, Investigation, Writing—review and editing. MK: Conceptualization, Methodology, Writing—original draft, Writing—review and editing.
